# Zika Virus Infection, Philippines, 2012

**DOI:** 10.3201/eid2104.141707

**Published:** 2015-04

**Authors:** Maria Theresa Alera, Laura Hermann, Ilya A. Tac-An, Chonticha Klungthong, Wiriya Rutvisuttinunt, Wudtichai Manasatienkij, Daisy Villa, Butsaya Thaisomboonsuk, John Mark Velasco, Piyawan Chinnawirotpisan, Catherine B. Lago, Vito G. Roque, Louis R. Macareo, Anon Srikiatkhachorn, Stefan Fernandez, In-Kyu Yoon

**Affiliations:** Philippines-AFRIMS (Armed Forces Research Institute of Medical Sciences), Cebu City, Philippines (M.T. Alera, J.M. Velasco, C.B. Lago);; University of Toronto, Toronto, Ontario, Canada (L. Hermann);; AFRIMS, Bangkok, Thailand (L. Hermann, C. Klungthong, W. Rutvisuttinunt, W. Manasatienkij, B. Thaisomboonsuk, P. Chinnawirotpisan, L.R. Macareo, S. Fernandez, I.-K. Yoon);; Cebu City Health Department, Cebu City (I.A. Tac-An, D. Villa);; Department of Health, Manila, Philippines (V.G. Roque, Jr.);; University of Massachusetts Medical School, Worcester, Massachusetts, USA (A. Srikiatkhachorn)

**Keywords:** Zika virus, Philippines, phylogeny, viruses

**To the Editor:** Zika virus (ZIKV), a mosquitoborne flavivirus, was first isolated from a rhesus monkey in Uganda in 1947 (*1*). This positive-sense, single-stranded RNA virus (family *Flaviviridae*, genus *Flavivirus*) has a 10,794-nt genome and is most closely related to Spondweni virus (*2*,*3*). Phylogenetic analyses have revealed 2 major lineages: Asian and African (*2*–*4*).

The first human infection with ZIKV was reported in Nigeria in 1954 (*5*). The virus caused only sporadic infections until 2007, when a large outbreak occurred on Yap, an island in the Federated States of Micronesia (*6*). In October 2013, ZIKV was detected in French Polynesia; since then, >400 laboratory-confirmed cases have been reported (*7*). ZIKV has spread across the South Pacific, and autochthonous cases have been reported in New Caledonia, Easter Island, and the Cook Islands. Several cases of ZIKV infections have been reported in travelers to Southeast Asia (*4*,*8*) and French Polynesia (*3*,*7*).

In March 2012, a prospective longitudinal cohort study, which included active surveillance for acute febrile illness, was initiated in Cebu City, Philippines (I. Yoon, unpub. data). Participants contacted study staff to report fever and were also contacted weekly by staff to determine if they had fever during the previous 7 days. Fever episodes triggered an acute-illness visit by a study nurse, who performed a clinical assessment of the patient and collected an acute-phase blood sample. During the first year of surveillance, 270 acute febrile illnesses were detected; 267 of the patients had samples available for serologic testing for evidence of influenza, dengue, chikungunya, Japanese encephalitis, and Zika virus infections.

In May 2012, a 15-year-old boy in Cebu City reported a subjective fever; an acute-illness investigation followed. Other symptoms included headache, conjunctivitis, sore throat, myalgias, stomach pain, anorexia, nausea, and vomiting, but no rash. The boy did not seek medical care or require hospitalization; his only treatment was acetaminophen. He had no recent travel history, and no other members of his household were ill. The boy recovered fully by the 3-week study follow-up visit. An acute-phase blood sample, collected 2 days after symptom onset, was negative for dengue and chikungunya viruses by reverse transcription PCR. An in-house dengue/Japanese encephalitis IgM/IgG capture ELISA and chikungunya ELISA were used to test paired acute- and convalescent-phase blood samples; all results were negative. ZIKV ELISA was not available at the testing laboratory. However, by using real-time reverse transcription PCR targeting the gene that encodes the precursor of membrane protein, we detected ZIKV RNA in the patient’s serum sample (*6*).

Virus was then isolated by intrathoracically inoculating *Toxorhynchites splendens* mosquitoes and by inoculating C6/36 cells with patient serum. The MiSeq platform (Illumina, Hayward, CA, USA) was used to obtain sequence reads by next-generation genomic sequencing, which identified a 789-bp contig as a partial sequence of the ZIKV gene that encodes the nonstructural 5 protein (GenBank accession no. KM851038).

Maximum-likelihood phylogenetic analysis of the gene encoding the nonstructural 5 protein sequence showed that the isolate belonged to the ZIKV Asian lineage (Figure). Pair-wise genetic distance calculation indicated that the isolate was most closely related to the 2007 strain from Micronesia (p-distance = 0.013), with which it shared ≈99% nt (779/789) similarity.

**Figure:**
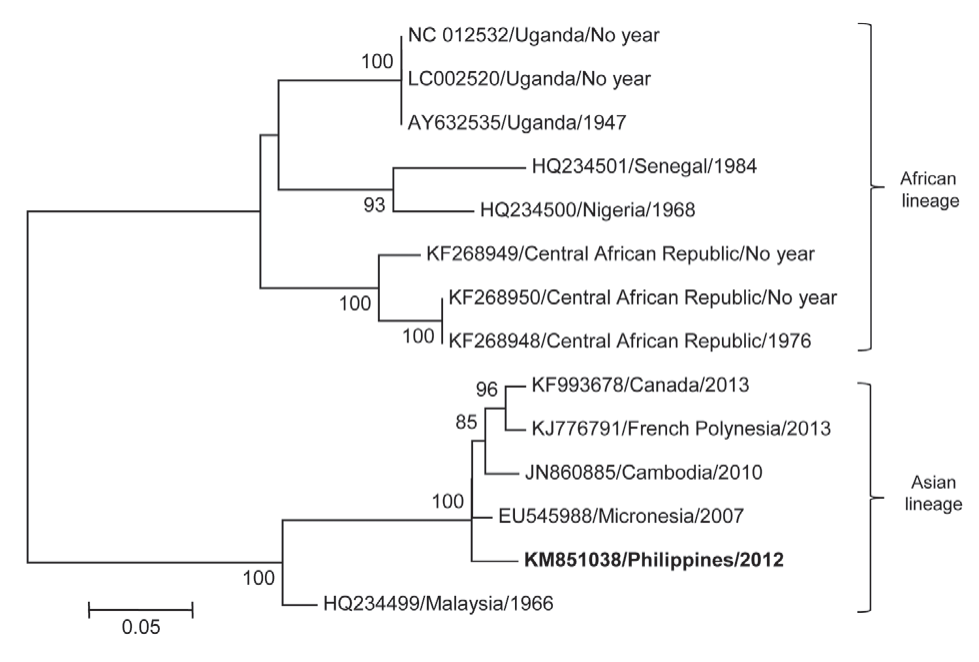
Maximum-likelihood phylogenetic tree of fragments of Zika virus was determined using the general time-reversible plus gamma distribution plus invariable site model with 13 reference Zika virus strains from GenBank. The contig sequence, obtained from de novo assembly and blastn (http://blast.ncbi.nlm.nih.gov/Blast.cgi?PROGRAM=blastn&PAGE_TYPE=BlastSearch&LINK_LOC=blasthome), of the Philippines isolate from 2012 (GenBank accession no. KM851038; bold font) was analyzed against 8 reference strains from Africa (GenBank accession nos. KF268948, KF268949, KF268950, LC002520, AY632535, NC012532, HQ234500, HQ234501) and 5 reference strains from Asia (GenBank accession nos. KJ776791, JN860885, EU545988, HQ234499, KF993678). The year of collection is unknown for several strains from Africa. Bootstrap values >70 are indicated at nodes. Scale bar indicates nucleotide substitutions per site. The drawing is not to scale.

During the past decade, ZIKV has caused 2 large epidemics in Micronesia and French Polynesia. The virus has a high potential for ongoing geographic expansion into countries where *Aedes* spp. mosquitoes are present and are known to transmit ZIKV; most notable among these vectors are *A. aegypti* mosquitoes, which are widespread throughout the Philippines (*9*). ZIKV infections have been reported in travelers to areas in the South Pacific with known ZIKV transmission and to areas such as Thailand (*4*) and Indonesia (*8*), where no recent endemic cases have been described. However, a case of endemic ZIKV infection has been reported in a child in Cambodia, and serologic evidence of ZIKV infection has been reported in Thailand, Vietnam, Malaysia, Indonesia, and the Philippines (*2*).

Phylogenetic analysis of the isolate from our study indicated that it is more closely related to the strain from Micronesia that was responsible for the 2007 Yap outbreak than to strains identified in Cambodia (2010), Thailand (2013), or French Polynesia (2013). It is possible that the ZIKV infection in our study was an isolated case (the virus was confirmed in only 1/270 episodes of acute febrile illness). However, because the symptoms of disease are similar to those for other known endemic arboviruses, it is also possible that the strain was introduced into the Philippines before 2012 and remained undetected.

The spectrum of ZIKV-associated clinical disease remains uncertain. Although reports indicate most cases of infection are mild, infections may be associated with more severe disease outcomes, such as Guillain-Barre syndrome (*10*). Increased surveillance for ZIKV disease, using pan-flavivirus or ZIKV-specific molecular testing, may lead to more frequent identification of cases, which could give a clearer indication of the true number of ZIKV infections. Additional surveillance and research studies are needed to improve our understanding of this disease, including the potential epidemiologic and clinical effects of ZIKV co-circulation with other flaviviruses.
